# Donor liver quality after hypovolemic shock and venous systemic oxygen persufflation in an experimental animal model

**DOI:** 10.1186/s40001-018-0346-5

**Published:** 2018-10-23

**Authors:** Azin Jafari, Hanno Matthaei, Vittorio Branchi, Edwin Bölke, Rene H. Tolba, Jörg C. Kalff, Steffen Manekeller

**Affiliations:** 1 Department of Surgery, Faculty of Medicine, Rheinische Friedrich-Wilhelms-Universität, Sigmund-Freudstr. 25, 53127 Bonn, Germany; 2 Department of Radiotherapy and Radiation Oncology, Faculty of Medicine, Heinrich-Heine-Universität, Düsseldorf, Germany; 3 Institute for Laboratory Animal Science and Experimental Surgery, University Hospital Rheinisch-Westfälische Technische Hochschule Aachen, Aachen, Germany

**Keywords:** Liver transplantation, Hypovolemic shock, Shock organs, VSOP

## Abstract

**Background:**

The ever growing demand for liver transplantation inevitably necessitates an expansion of the donor pool. Utilization of “shock organs” is considered suboptimal to date while the associated outcome has hardly been investigated.

**Materials and methods:**

Male Wistar rats underwent a period of 30 min of hypovolemic shock. After 24 h livers were explanted and prior to reperfusion underwent either 18 h of cold storage (CS; *N* = 6) or 17 h of CS followed by 60 min venous systemic oxygen persufflation (VSOP; *N* = 6). The outcome of “shock organs (SHBD)” was compared to heart-beating donor (HBD; *N* = 12) as positive control and non-heart-beating donor (NHBD; *N* = 12) as negative control animal groups. Liver function was assessed by measuring enzyme release (AST, ALT, LDH), bile production, portal vein pressure and hepatic oxygen uptake during reperfusion. For reperfusion, the isolated perfused rat liver system was used.

**Results:**

Liver function was severely limited in NHBD group compared to HBD organs after 18 h of CS (e.g., AST; HBD: 32.25 ± 7.25 U/l vs. NHBD: 790 ± 414.56 U/l; *p* < 0.005). VSOP improved liver function of NHBD organs significantly (AST; NHBD + VSOP: 333.6 ± 149.1 U/l; *p* < 0.005). SHBD organs showed a comparable outcome to HBD and clearly better results than NHBD organs after 18 h of CS (AST; SHBD: 76.4 ± 21.9 U/l). After 17 h of CS accompanied by 60 min VSOP, no improvement concerning liver function and integrity of SHBD organs was observed while the results were severely deteriorated by VSOP resulting in higher enzyme release (AST; SHBD + VSOP: 213 ± 61 U/l, *p* < 0.001), higher portal vein pressure (SHBD: 10.8 ± 1.92 mm Hg vs. SHBD + VSOP: 21.6 ± 8.8 mm Hg; *p* < 0.05) and lower hepatic oxygen uptake (SHBD: 321.75 ± 3.87 ml/glw/min vs. SHBD + VSOP: 395.8 ± 46.64 ml/glw/min, *p* < 0.05) at 24 h.

**Conclusions:**

Our data suggest that the potential of “shock organs” within liver transplantation may be underestimated. If our findings are reproducable in humans, SHBD grafts should be considered as a valuable source for expanding the thus far limited donor pool.

## Introduction

Since liver transplantation has become the most effective therapy in end-stage liver disease and the demand of suitable organs continues to increase, the main focus of liver transplant research is the expansion of the donor pool. Thus far, a majority of liver transplants stem from donations after brain death. However, due to a shortage of young and healthy organs one possible approach is the use of “less than optimal” grafts from donors fulfilling the extended donor criteria [1–4]. Those include the potentially compromising factors: higher age of the donor, duration of intensive care stay and mechanical ventilation > 6 days, body mass index > 30 kg/m^2^, prolonged ischemia, hepatic steatosis, viral hepatitis, and certain causes of donor death [5]. Another option is represented by organ donation after cardiac death, referred to as “non-heart-beating donors (NHBD)” [6–8]. In fact, before the establishment of the Harvard definition of brain death liver transplantation was performed using grafts from cardiac death patients with dismal outcome. While therefore largely declined in the beginnings of transplant history, NHBD organs have increasingly been transplanted in the past years as “marginal organs” with acceptable results [9, 10]. Later in the nineties, organs from brain dead donors were increasingly and successfully used contrasting these poor results from in the meantime only rarely used NHBD grafts [11, 12]. With an ever growing demand and procedural optimization, a renaissance of NHBD organs could be observed. Especially, controlled cardiac death liver transplantation, legally permitted, has been a valuable transplant source throughout the recent years. These organs culminate in up to 20% in the Dutch liver graft pool whereas in Germany this option is still forbidden owing to higher graft failure compared to organs from brain dead donors [13].

A third solution for expanding the donor pool is the utilization of organs from brain death donors after surviving a hypotensive period through successful resuscitation. Though these so-called “shock heart-beating donor” (SHBD) organs are also considered as marginal [14], they have been successfully used in small patient cohorts in lung transplantation [15], heart transplantation [16], intestinal transplantation [17], and also liver transplantation [18–20]. In spite of these encouraging preliminary results, SHBD transplantation remains quite controversial. In particular, with respect to primarily liver dysfunction and non-function and the scarcity of experimental and clinical data, this option is till today not fully appreciated in the transplant community. Additionally, the possibility of optimizing “shock organs” with various already existing techniques is not sufficiently investigated.

Thus, with the present study we sought to examine the outcome of “shock organs” in an isolated perfused rat liver (IPRL) by mimicking severe hypotension prior to liver donation. In a second step, the possibility of optimizing SHBD organs by venous systemic oxygen persufflation (VSOP), as it has been already applied successfully with NHBD organs, was explored.

## Materials and methods

### Animals

Animal experiments were performed in accordance with the federal German law regarding the protection of animals. The principles of laboratory animal care were followed (NIH Publication No. 85-23, revised 1985). In all experiments, male Wistar rats, obtained from Charles River Laboratories, weighing 200–250 g, were used as liver donors.

## Experimental design

### Control groups

Heart-beating donors (HBD) were defined as positive control and non-heart-beating donors (NHBD) were regarded as negative control. General anesthesia was induced by inhalation of isoflurane (Abbott GmBH & Co. KG, Wiesbaden, Germany). Midline laparotomy with bilateral subcostal extensions was performed and the liver was sceletonized and freed from all ligamentous attachments. For bile collection, the common bile duct was cannulated with a 0.3 × 0.6 mm polytetrafluoroethylene tube (Sigma-Aldrich Inc., St. Louis, USA). After hepatic artery ligation, the portal vein was cannulated with a 14-gauge polyethylene catheter (B. Braun Melsungen AG, Melsungen, Germany) for perfusion with 20 ml 0.9% saline solution (B-Braun Melsungen AG, Melsungen, Germany). To prevent hepatic outflow obstruction, the inferior caval vein was incised. After final liver explantation perfusion with 60 ml histidine tryptophane ketoglutarate (HTK) solution with 20 mM *N*-acetylcysteine (NAC, Hexal AG, Holzkirchen, Germany) was performed at 4 °C and an additional 14-gauge catheter inserted into the supra hepatic caval vein for following reperfusion. Finally, livers were stored in 125 ml HTK at 4 °C with a cold water bath (Ministat 125, Peter Huber Kältemaschinenbau GmbH, Offenburg, Germany) for 17 h (*N* = 6 animals, HBD + VSOP) and, respectively, 18 h (*N* = 6 animals, HBD).

In the NHBD groups (NHBD + VSOP with *N* = 6 animals, NHBD with *N* = 6), cardiac arrest was induced by phrenotomy for 30 min consequently leading to warm ischemia before portal vein cannulation and liver explantation.

### Study group

In the shock heart-beating donor groups (SHBD with *N* = 6 and SHBD + VSOP with *N* = 6), animals underwent a period of 30 min of hypotension 24 h prior to liver explantation, in accordance with the protocol of the HBD group. For hypotension induction, the fixed-volume hemorrhage model was applied [21] and general anesthesia was performed as described above. The right carotid artery was dissected and cannulated with a polyethylene catheter (PE 50) and connected to a high sensitivity transducer (Capto SP 844 Physiologic Pressure Transducer, Capto Inc., Skoppum, Norway) for the measurement of mean and systolic arterial pressure. Afterwards, the right femoral vein was also dissected and cannulated with a polyethylene catheter and 30% of blood volume was drawn. The consequently following hypotension was maintained for 30 min. Subsequently, animals were injected with 0.9% saline solution according to the equivalent volume of their blood loss. After securing hemodynamic stability, catheters were removed and the blood vessels ligated [22].

### VSOP

All groups of animals were further divided into cohorts undergoing either 17 or 18 h of cold storage. Livers with 17 h of cold storage (+VSOP groups) were persufflated with medical-grade gaseous oxygen for another 60 min prior to reperfusion as described before [23]. In brief, the catheterized superior caval vein was used for oxygen persufflation at a pressure of 18 mmHg. The margins of each liver lobe were punctuated with fine acupuncture needles (0.18 × 0. 30 mm, Seirin Corp., Shizuoka, Japan) in order to eliminate gas and to prevent damage to the liver microvasculature.

### Reperfusion

Prior to reperfusion, each liver was warmed up for 30 min at 24 °C to simulate rewarming during reimplantation. The isolated perfused rat liver system was used as described before [24]. In summary, the perfusion circuit was prerinsed with 200 ml of a saline solution and afterwards rinsed with 100 ml of Krebs–Henseleit buffer (Sigma-Aldrich Co., St. Louis, USA) modified by additional application of calcium chloride (Sigma-Aldrich Co., St. Louis, USA) and sodium hydrogen carbonate (Fresenius Kabi Deutschland GmbH, Bad Homburg, Germany). Finally, reperfusion for 60 min was performed in a recirculating system at a constant flow of 3 ml/g of liver weight per minute with a special roller pump (Masterflex L/S, Cole-Parmer Instrument Co., Vernon Hills, Illinois) with 220 ml of oxygenated modified Krebs–Henseleit buffer at 37°. Oxygenation and perfusate pO2 were maintained at a minimum of 500 mmHg during the reperfusion period as measured by blood gas analysis (ABL 5, Radiometer, Copenhagen, Denmark).

### Enzyme release

After 5, 15, 30, 45 and 60 min hepatic effluent was collected and analyzed for the release of specific liver enzymes including alanine transaminase and aspartate transaminase (AST, ALT) and lactate dehydrogenase (LDH) to quantify the extent of liver injury. The examination was performed by a standard enzymatic method with a Vitros 250 analyzer (Ortho-Clinical Diagnostics, Raritan, NJ).

### Bile production

The common bile duct was cannulated as described above and bile production per time measured during reperfusion. The extent of bile production should thereby serve as an indicator for the functional capacity of the reperfused liver.

### Portal vein pressure (PVP)

Portal vein pressure was continuously measured by a water column connected to the portal vein inflow catheter. The measuring system was calibrated at the start of each reperfusion procedure. PVP measurements were performed to evaluate vascular resistance as another parameter of liver injury.

### Hepatic oxygen uptake

Oxygen concentration of portal inflow and venous effluent was measured by perfusate samples with the ABL 5 blood gas analyser (Radiometer A/S, Copenhagen, Denmark). The difference between portal and central venous oxygenation was determined as oxygen uptake and expressed as microliters per gram of liver weight per minute.

### Statistics

Statistical analyses were performed using Graph Pad Prism v. 5 (GraphPad Software, Inc., San Diego, CA, USA). Data are expressed as mean ± SD. Differences in the measured variables between each group were assessed using one-way Anova or two-way Anova. *p* < 0.05 was considered to indicate statistical significance.

## Results

### Liver enzyme levels

**Fig. 1:**
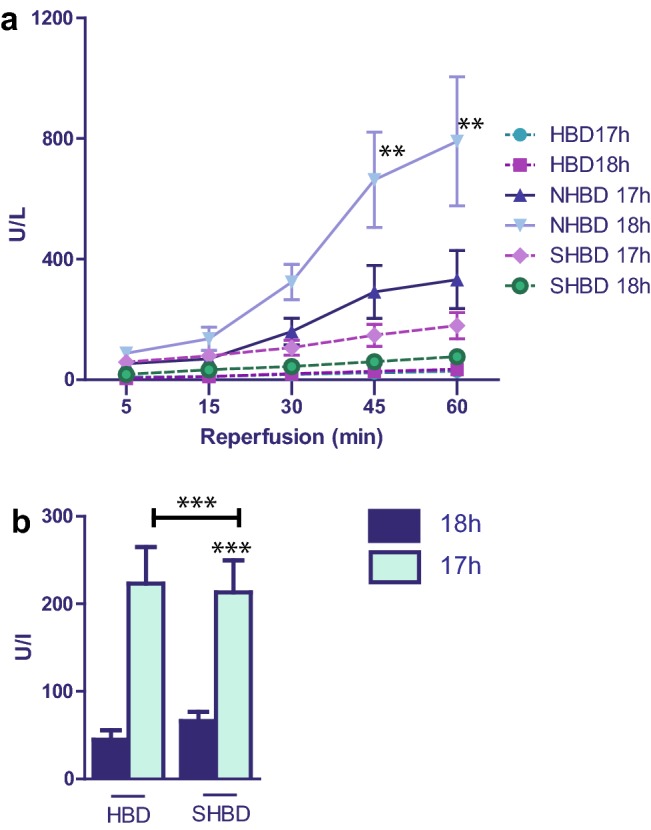
Result of AST release in hepatic effluent. SHBD group: Animals underwent a period of 30 min of hypovolemic shock. 24 h later livers were explanted and prior to reperfusion underwent either 18 h of CS (SHBD, *N* = 6) or 17 h of CS with 60 min VSOP (SHBD + VSOP, *N* = 6). NHBD group: Prior to liver explantation cardiac arrest was induced by phrenotomy for 30 min. Reperfusion was performed either after 18 h of CS (NHBD; *N* = 6) or 17 h of CS with 60 min VSOP (NHBD + VSOP, *N* = 6). NHBD served as a negative control group. HBD group: Liver explantation was performed under heart-beating conditions. Reperfusion was performed either after 18 h of CS (HBD; *N* = 6) or 17 h of CS with 60 min VSOP (HBD + VSOP, *N* = 6). NHBD served as negative control group. During reperfusion, hepatic effluent was collected at 5, 15, 30, 45 and 60 min and enzyme release measured. **a** Results of all six groups are shown. **b** For a better overview, only the results of HBD and SHBD groups are depicted. Major significances are shown; *p < 0.05; **p < 0.005; ***p < 0.001; ****p < 0.0001

**Fig. 2:**
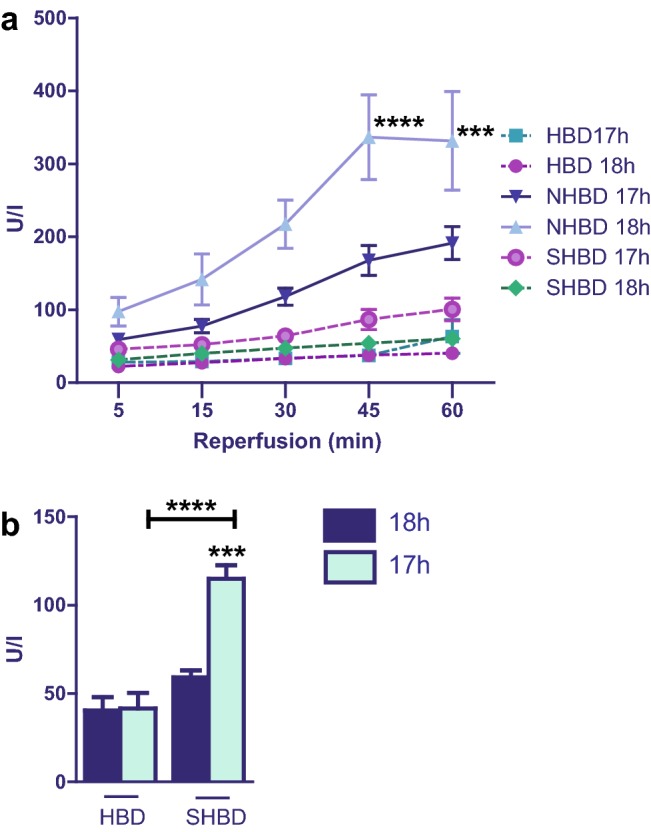
Result of ALT release in hepatic effluent. SHBD group: Animals underwent a period of 30 min of hypovolemic shock. 24 h later livers were explanted and prior to reperfusion underwent either 18 h of CS (SHBD, *N* = 6) or 17 h of CS with 60 min VSOP (SHBD + VSOP, *N* = 6). NHBD group: Prior to liver explantation, cardiac arrest was induced by phrenotomy for 30 min. Reperfusion was performed either after 18 h of CS (NHBD; *N* = 6) or 17 h of CS with 60 min VSOP (NHBD + VSOP, *N* = 6). NHBD served as a negative control group. HBD group: Liver explantation was performed under heart-beating conditions. Reperfusion was performed either after 18 h of CS (HBD; *N* = 6) or 17 h of CS with 60 min VSOP (HBD + VSOP, *N* = 6). HBD served as positive control group. During reperfusion, hepatic effluent was collected at 5, 15, 30, 45 and 60 min and enzyme release measured. **a** Results of all six groups ar shown. **b** For a better overview, only the results of HBD and SHBD groups are depicted. Major significances are shown; *p < 0.05; **p < 0.005; ***p < 0.001; ****p < 0.0001

Between HBD + VSOP and HBD, no difference regarding the GOT level was observed (Fig. 1a, b). In the NHBD group, animals with VSOP showed significantly lower AST levels compared to those livers subjected to cold storage only (NHBD, Fig. 1a). SHBD livers with VSOP, however, did not benefit from VSOP. Even more, VSOP resulted in significantly higher AST levels compared to mere cold storage (SHBD; Fig. 1b). Overall, the lowest enzyme levels were seen in the HBD groups and the highest levels in the NHBD groups (Fig. 1a). Release of AST in SHBD group did not significantly differ from the result in the HBD cohort, whereas with VSOP SHBD lead to significantly higher hepatocellular damage and thus AST release compared to HBD. Accordingly, results were obtained from ALT measurements (Fig. 2a, b).

### LDH level

**Fig. 3:**
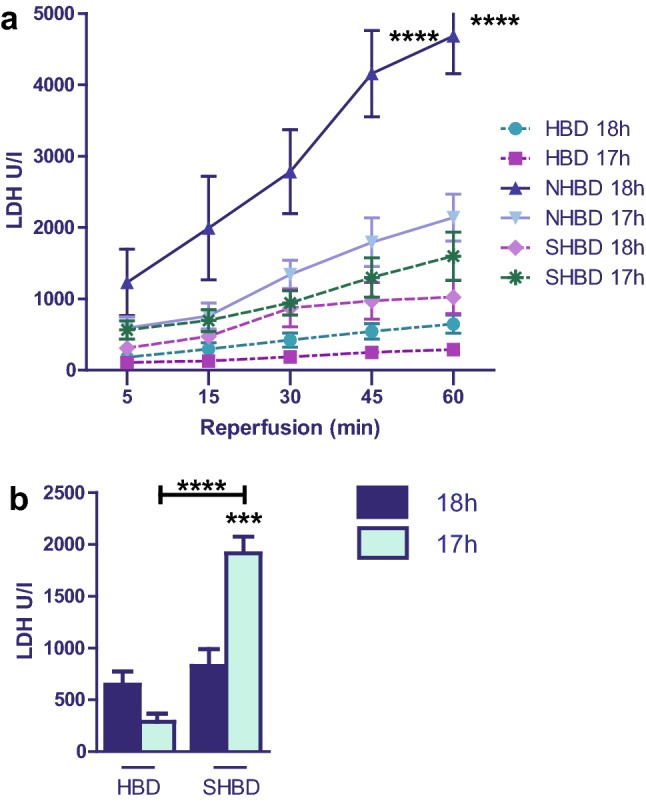
Result of LDH release in hepatic effluent. SHBD group: Animals underwent a period of 30 min of hypovolemic shock. 24 h later livers were explanted and prior to reperfusion underwent either 18 h of CS (SHBD, *N* = 6) or 17 h of CS with 60 min VSOP (SHBD + VSOP, *N* = 6). NHBD group: Prior to liver explantation cardiac arrest was induced by phrenotomy for 30 min. Reperfusion was performed either after 18 h of CS (NHBD; *N* = 6) or 17 h of CS with 60 min VSOP (NHBD + VSOP, *N* = 6). NHBD served as a negative control group. HBD group: Liver explantation was performed under heart-beating conditions. Reperfusion was performed either after 18 h of CS (HBD; *N* = 6) or 17 h of CS with 60 min VSOP (HBD + VSOP, *N* = 6). HBD served as positive control group. During reperfusion, hepatic effluent was collected at 5, 15, 30, 45 and 60 min and enzyme release measured. **a** Results of all six groups ar shown. **b** For a better overview only the results of HBD and SHBD groups are depicted. Major significances are shown; *p < 0.05; **p < 0.005; ***p < 0.001; ****p < 0.0001

Correlating to the results above, VSOP showed no influence in the HBD groups. However, a highly positive influence on the NHBD + VSOP cohort with a significantly milder enzyme release was observed (Fig. 3a). SHBD organs in contrast were deteriorated by VSOP leading to significantly higher enzyme release in the SHBD + VSOP group compared to cold storage only (SHBD; Fig. 3a, b). In general, NHBD organs showed the highest enzyme levels, followed by the SHBD + VSOP cohort. The LDH levels in both HBD groups and in the SHBD groups were not noteworthy elevated.

### Bile production

**Fig. 4:**
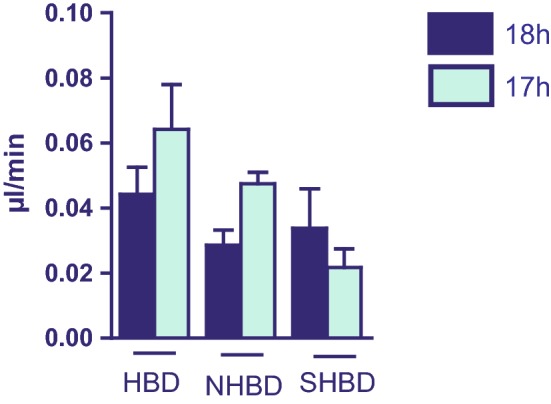
Measurement of bile production during reperfusion. SHBD group: Animals underwent a period of 30 min of hypovolemic shock. 24 h later livers were explanted and prior to reperfusion underwent either 18 h of CS (SHBD, *N* = 6) or 17 h of CS with 60 min VSOP (SHBD + VSOP, *N* = 6). NHBD group: Prior to liver explantation, cardiac arrest was induced by phrenotomy for 30 min. Reperfusion was performed either after 18 h of CS (NHBD; *N* = 6) or 17 h of CS with 60 min VSOP (NHBD + VSOP, *N* = 6). NHBD served as negative control group. HBD group: Liver explantation was performed under heart-beating conditions. Reperfusion was performed either after 18 h of CS (HBD; *N* = 6) or 17 h of CS with 60 min VSOP (HBD + VSOP, *N* = 6). HBD served as positive control group. In all animals, the common bile duct was cannulated and bile production was measured throughout reperfusion. The total amount of bile after 60 min of reperfusion was used for calculation. Major significances are shown; *p < 0.05; **p < 0.005; ***p < 0.001; ****p < 0.0001

There were no statistic differences measured regarding bile production among all groups. A slight trend was observed with respect to VSOP which seems to improve the functional liver capacity in HBD and NHBD livers compared to 18 h of CS. However, SHBD livers tended to a decreased bile production subsequent to VSOP administration (Fig. 4).

### PVP

**Fig. 5:**
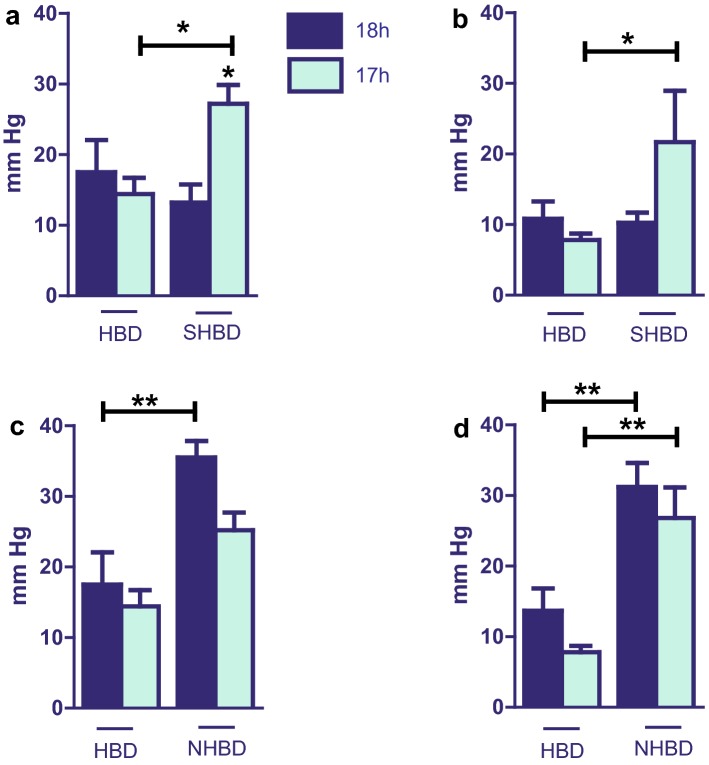
Portal vein pressure. SHBD group: Animals underwent a period of 30 min of hypovolemic shock. 24 h later livers were explanted and prior to reperfusion underwent either 18 h of CS (SHBD, *N* = 6) or 17 h of CS with 60 min VSOP (SHBD + VSOP, *N* = 6). NHBD group: Prior to liver explantation, cardiac arrest was induced by phrenotomy for 30 min. Reperfusion was performed either after 18 h of CS (NHBD; *N* = 6) or 17 h of CS with 60 min VSOP (NHBD + VSOP, *N* = 6). HBD served as positive control group. HBD group: Liver explantation was performed under heart-beating conditions. Reperfusion was performed either after 18 h of CS (HBD; *N* = 6) or 17 h of CS with 60 min VSOP (HBD + VSOP, *N* = 6). NHBD served as negative control group. Portal venous pressure was measured during isolated perfusion by means of a water column connected to the portal inflow line and precalibrated to the calculated flow of 3 ml/g/min using. Total vascular resistance was calculated from transhepatic flow and portal perfusion pressure. **a** Time point 5 min after reperfusion: HBD vs. SHBD. **b** Time point 60 min after reperfusion: HBD vs. SHBD. **c** Time point 5 min after reperfusion: HBD vs. NHBD. **d** Time point 60 min after reperfusion: HBD vs. NHBD. Major significances are shown; *p < 0.05; **p < 0.005; ***p < 0.001; ****p < 0.0001

In order to focus our results, only the time points 5 and 60 min are depicted in Fig. 5. Vascular resistance was not significantly influenced by VSOP among HBD groups and NHBD groups. Although, a tendency towards lower pressures after VSOP can be noticed in both groups. Compared to HBD, NHBD organs showed a significantly higher vascular resistance. Once again, VSOP with shock organs resulted in higher PVP. At 5 min, a statistical significance between SHBD + VSOP and SHBD could be observed (Fig. 5a). Shock induction and cold storage (SHBD) did not increase PVP compared to controls.

### Hepatic oxygen uptake

**Fig. 6:**
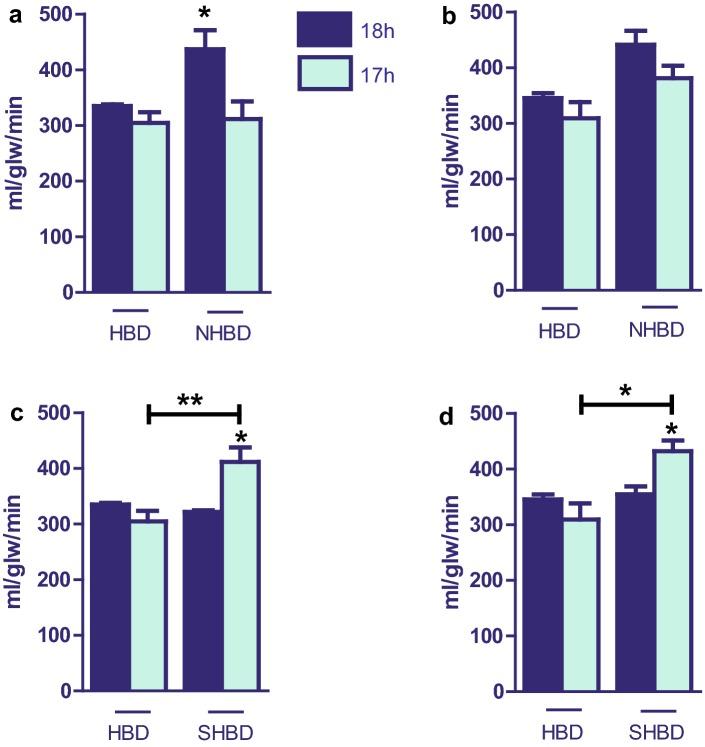
Hepatic oxygen uptake. SHBD group: Animals underwent a period of 30 min of hypovolemic shock. 24 h later livers were explanted and prior to reperfusion underwent either 18 h of CS (SHBD, *N* = 6) or 17 h of CS with 60 min VSOP (SHBD + VSOP, *N* = 6). NHBD group: Prior to liver explantation cardiac arrest was induced by phrenotomy for 30 min. Reperfusion was performed either after 18 h of CS (NHBD; *N* = 6) or 17 h of CS with 60 min VSOP (NHBD + VSOP, *N* = 6). NHBD served as negative control group. HBD group: Liver explantation was performed under heart-beating conditions. Reperfusion was performed either after 18 h of CS (HBD; *N* = 6) or 17 h of CS with 60 min VSOP (HBD + VSOP, *N* = 6). HBD served as positive control group. Perfusat samples were taken at the portal inflow and from the venous effluent and oxygen content was measured immediately in a pH–blood gas analyser. Oxygen uptake was calculated from the differences between portal and venous sites and expressed according to transhepatic flow and liver mass. **a** Time point 5 min after reperfusion: HBD vs. NHBD. **b** Time point 60 min after reperfusion: HBD vs. NHBD. **c** Time point 5 min after reperfusion: HBD vs. SHBD. **d** Time point 60 min after reperfusion: HBD vs. SHBD. Major significances are shown; *p < 0.05; **p < 0.005; ***p < 0.001; ****p < 0.0001

Examining oxygen uptake at time point 5 min after reperfusion, there was no difference in HBD groups. In contrast, in NHBD cohorts VSOP significantly improved oxygen metabolism (Fig. 6a). At 60 min, a similar tendency could be seen not quite reaching statistical significance (Fig. 6b). In SHBD groups, VSOP significantly impaired oxygen uptake at 5 and 60 min. The results in group SHBD + VSOP are comparable to those in NHBD (Fig. 6c, d).

## Discussion

During the past decades, liver transplantation has experienced significant advances regarding donor management, graft preparation, surgical technique, perioperative anaestesiologic and intensive care treatment, as well as immunosuppression. Due to these improvements, this complex, costly and invasive therapy remains undoubtedly the most important therapeutic component in end-stage liver disease. Hence, waiting lists for liver transplantation are growing rapidly worldwide whereas, unfortunately, the allocation of liver grafts remains static at best. This problem has been approached in various ways. For example, split-liver techniques are advocated in order to serve two recipients from one liver graft [25]. Living donor programs have been successfully established, especially in children [26]. Furthermore, liver tissue engineering is another vital scientific field to hopefully address organ shortage in the near future [27].

Nonetheless, the remaining gap between organ demand and availability forces the transplant community to identify more alternatives to balance this prevailing asymmetry. Formerly neglected “less than optimal organs” are increasingly moving into focus including attempts to optimize these by improving “ex vivo” preservation. Extended graft criteria lead to accepting marginal organs including steatotic grafts or livers from elderly patients. The combination of these methods has caused an increasing use of NHBD organs in spite of their inferior outcome concerning ischemia reperfusion injury compared to optimal healthy grafts in the past few years [6, 8, 28–32]. Since organ shortage will nevertheless persist, we focussed on SHBD grafts as yet another transplant opportunity and additionally assessed the option of VSOP. The idea of transplanting shock organs is not entirely new although functional damage by hypoxia and subsequent patient resuscitation have been accused to possibly impair future organ function: why clinical and experimental data on this topic are rare. Elaffandy et al. compared in a prospectively collected database the outcome of grafts from donors with prehospital cardiac arrest with organs from donation after circulatory death. Liver donation with a history of prehospital cardiac arrest was accepted if transaminase levels were ≤ 4 times the normal range and presenting an improving trend. Interestingly, the authors found no significant difference in graft or patient survival with even better short-term results for organs with prehospital cardiac arrest [33]. Faucher et al. reported in a descriptive study about a series of successful organ transplantations of donor grafts with out-of-hospital traumatic cardiac arrest [34]. Though the case number was small (nine donors with out-of hospital traumatic cardiac arrest), the results are remarkable. From nine donors, 31 organs were transplanted and showed no functional losses after 1 year. Still, the functional capacity and safety of these organs need further elucidation in order to safely add SHBD grafts into the donor pool.

While no standard protocol has been established for producing “shock” livers in animals, there are three predominant models used in hemorrhagic shock studies, thus far comprising fixed-volume hemorrhage, fixed-pressure hemorrhage and uncontrolled hemorrhage [35]. We chose the fixed-volume hemorrhage model in which a predetermined amount of the calculated blood volume is removed over a certain time period. This method is widely used in the studies with shock-induced experiments as it is more accessible and better reproducible.

Aside from the quality of the organ itself, the type of *post-mortem* preservation is crucial for successful outcome in transplantation. Herein, two distinct techniques have emerged as particularly supportive in the past years: On the one hand, hypothermic machine perfusion has been studied for decades in animal models. A limitation of ischemia/reperfusion injury through hypothermia has similarly been evidenced in humans according to a first clinical series reported by Guarrera et al. [36]. The same group of investigators suggested that on a molecular level hypothermic machine perfusion may lead to interruption of acute-phase inflammation protein secretion in the graft potentially attenuating reperfusion-related graft damage [37]. The promising benefits do, however, not only derive from stabilizing the microvasculature since hypothermic machine perfusion also incorporates the ability to deliver drugs to the ex vivo graft. Furthermore, it leads to dilution of harmful metabolites produced by anaerobic metabolism. Minor et al. pioneered in the field of VSOP research and established an animal model of aerobic ischemia in Wistar rats for subsequent scientific work in this very field in 1996 [38]. Ever since, VSOP has proven to benefit liver function while reducing the risk of primary organ dysfunction, especially with regard to NHBD grafts [23, 24, 29, 39, 40]. Encouraged by these promising results, the OPAL trial was initiated wherein oxygen persufflation as adjunct in liver preservation will be investigated in a prospective single-center randomized proof of concept study. First data from this trial are soon to be expected [41].

Hence, in the presented study we used VSOP as an optimizing tool since this method is technically less demanding and relatively straightforward to implement.

From our results, it can be derived that, as previously observed, HBD organs with 18 h of cold storage have a much better liver function than NHBD organs. SHBD grafts experienced a similar outcome to HBD organs after 18 h of cold storage. This suggests that utilization of organs that have suffered a limited period of hypotension may be safe. While our chosen hypotensive interval of 30 min may have been too brief for a measurable clinical impact in the rat liver the damage became more apparent in VSOP groups of rats. In the negative control group (NHBD), VSOP reduced liver damage and improved functional capacity. Interestingly, however, SHBD organs were significantly deteriorated by VSOP. The attempt to optimize “shock livers” with oxygen persufflation resulted, at least according to our scheduled measurements, in higher enzyme release, less bile production, rise of vascular resistance and lower hepatic oxygen uptake. It remains elusive, if this is a true more severe impact or if our results are merely a metabolic snapshot in a prolonged recovery phase of VSOP SHBD organs. This possible time dependency should be explored since a longer interval could lead to even more favorable results.

## Conclusion

While there is only minor hope for effective alternative treatment perspectives in the end-stage liver disease, liver transplantation will remain the only way for curing patients from hepatic failure. The growing demand for donor grafts forces the transplant community to explore the boundaries of graft acceptance at best without rising the risks for recipients. The presented results suggest that under defined circumstances the outcome of “shock organs” may correlate with the fate of HBD grafts. VSOP appears to deteriorate “shock liver” graft function while long-term observation shall more precisely elucidate the organ performance during recovery process. Therefore, the promising option of using “shock organs” to expand the donor pool needs further investigation.

## Abbreviations

CS: cold storage
VSOP: venous systemic oxygen persufflation
SHBD: shock heart-beating donors
NHBD: non-heart-beating donors
HBD: heart-beating donors
ALT: alanine transaminase
AST: aspartate transaminase
LDH: lactatdehydrogenase
IPRL: isolated perfused rat liver
HTK: histidine tryptophane ketoglutarate
PVP: portal vein pressure
